# Supplementary material for: Schools are segregated by educational outcomes in the digital space
Source: PLoS One. 2019 May 28;14(5):e0217142. doi: 10.1371/journal.pone.0217142 (PMC6538368; doi:10.1371/journal.pone.0217142)
Supplement: S1 Text — (PDF) [file pone.0217142.s001.pdf]

Supplementary Information for "Schools are segregated by educational outcomes in the digital space"

Ivan Smirnov[1*]

1 Institute of Education, National Research University Higher School of Economics, Moscow, Russia

* ibsmirnov@hse.ru

Details on the Russian school system

The Russian education system is a progeny of the Soviet system that was characterized by its egalitarian nature and high level of standardization. Children start their compulsory education at the age of 6-8 in Russia. The first 9 years of studies are mandatory. A significant number of students continue their education for two more years. Entrance tests to the first grade in schools are forbidden by law. Instead, admission to a school is based on how close a child lives to it. As a general rule, each residential address is assigned to a certain school. Most of the Russian schools are public, less than 1% of Russian students attend private school [1].

The situation is more nuanced for higher grades. There are several so-called "magnet schools" in Saint Petersburg. These are high performing selective schools that attract students from all over the city. Most transitions to these schools from other schools take place during the last school years as parents want their children to be better prepared for the final exams. Our sample does not include students from the last grades. However, the presence of such schools might partially explain why high performing schools are also among the most central in the network.

VK sample and potential bias

VK requires all users to indicate their age however the school number is not required. It means that there are two potential sources of bias in our sample. First, not everyone is registered on VK. Note, however, that VK is ubiquitous among young Russians. More than 93% of 18–24 years old use it regularly [2]. This number is probably even higher for large cities like Saint Petersburg. It means that only a small fraction of students are not registered on VK and given the fact that our study is about online connections, this potential bias should not be relevant for our results.

Second, our sample is constructed in such a way that it includes only those users who indicate their school in profile. There is some limited evidence that there is no difference in age, gender or academic performance between students who indicate their school in profile and those who do not [3]. While there is no evidence for substantial bias in our sample it is still not random. This might have some implications for the interpretation of the results. For instance, the level of activity on VK might be correlated with academic performance. In this case, the students from higher performing schools might be more active in creating connections and it might explain the central position of these schools in the network. The observed homophily in academic performance would not be affected by such bias.

References

1. National Research University Higher School of Economics. Indicators of education; 2018. https://www.hse.ru/primarydata/io2018.
2. Public Opinion Foundation. Online practices of Russians: social networks; 2016. http://fom.ru/SMI-i-internet/12495.
3. Smirnov I, Sivak E, Kozmina Y. In Search of Lost Profiles: The Reliability of VKontakte Data and its Importance in Educational Research. Educational Studies. 2016;4:106–122.
