# Supplementary figures and images for: Schools are segregated by educational outcomes in the digital space
Source: PLoS One. 2019 May 28;14(5):e0217142. doi: 10.1371/journal.pone.0217142 (PMC6538368; doi:10.1371/journal.pone.0217142)

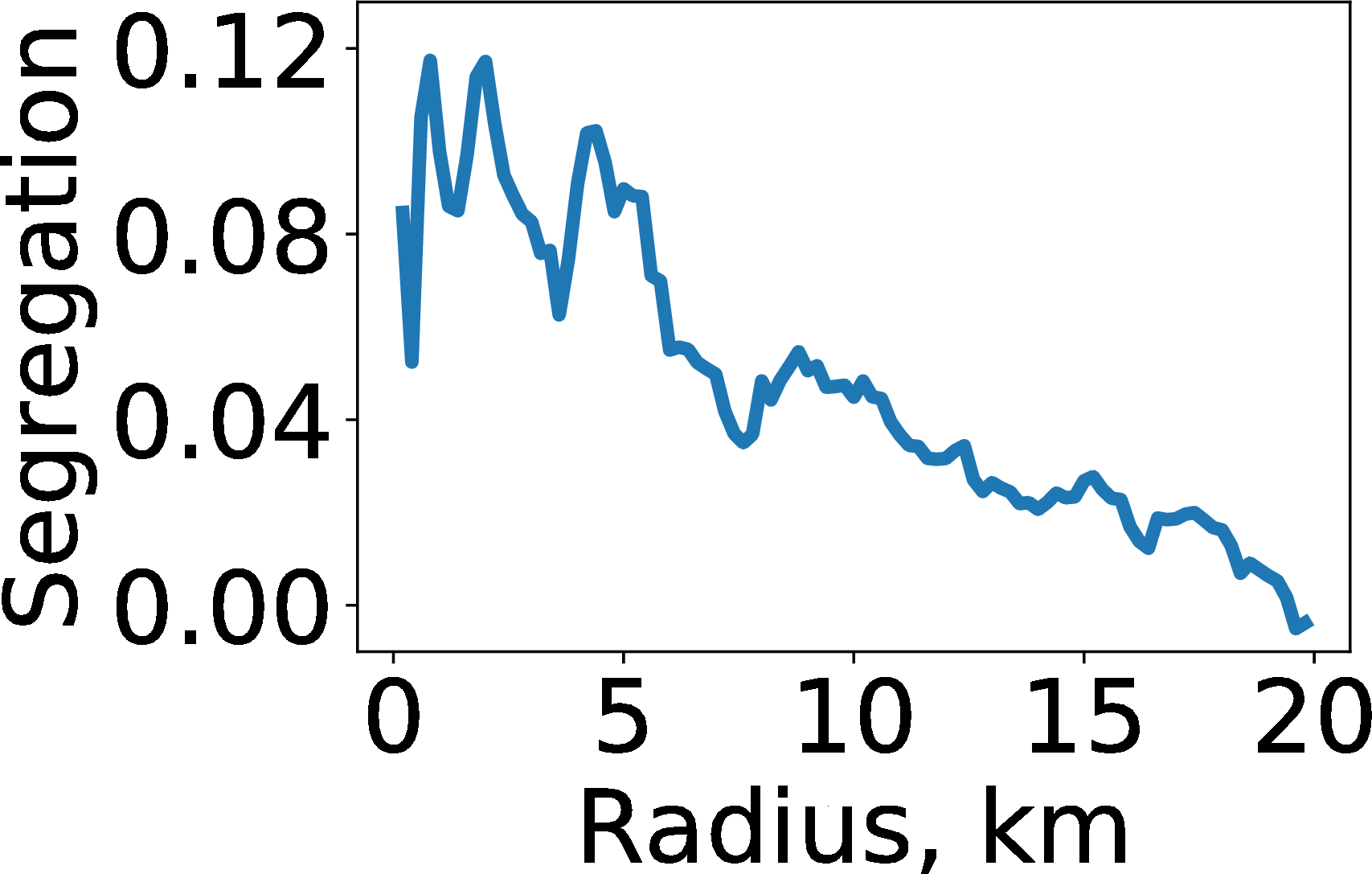

Supplement: S1 Fig — (TIF) [file pone.0217142.s002.tif]

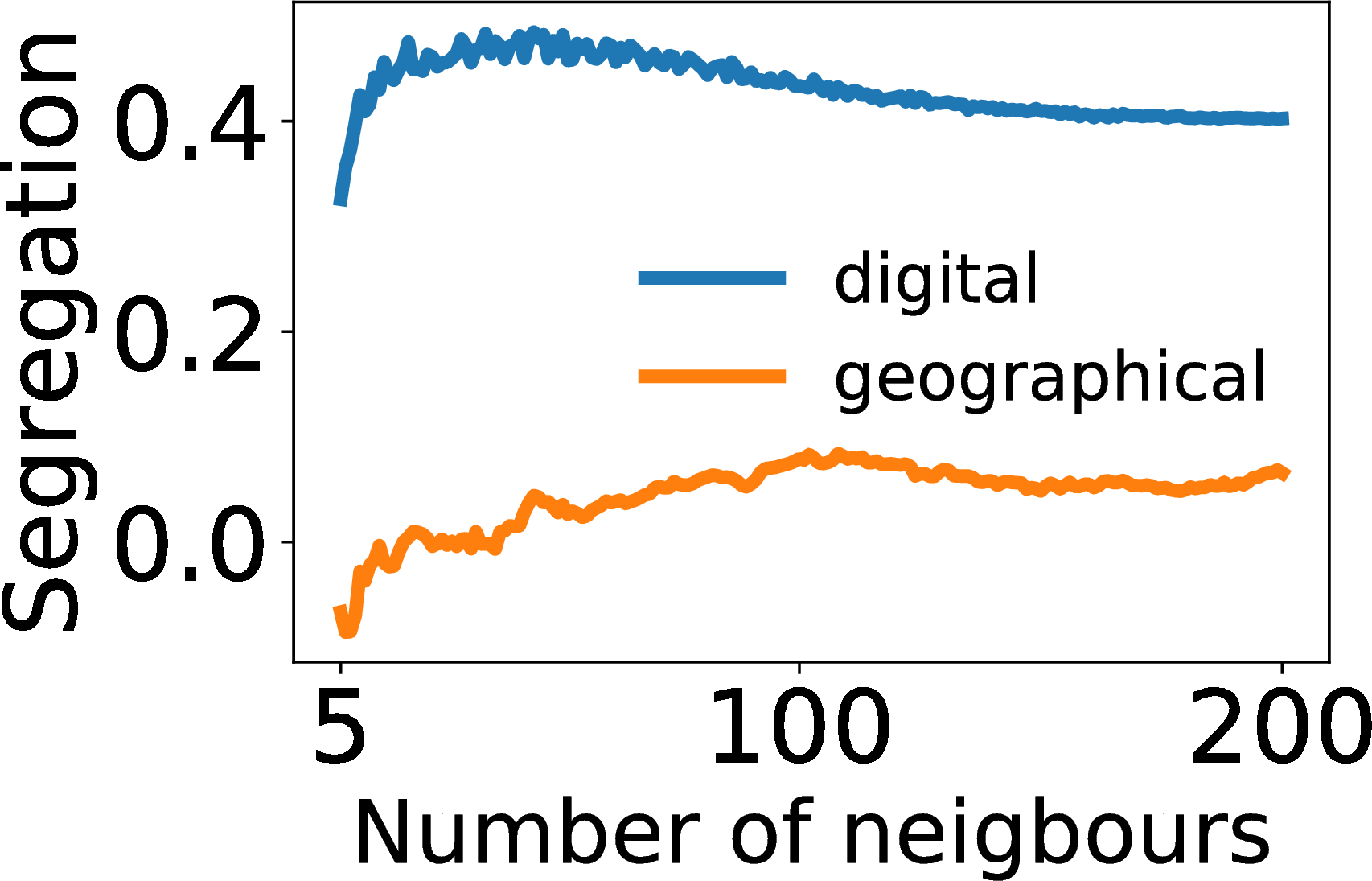

Supplement: S2 Fig — (TIF) [file pone.0217142.s003.tif]

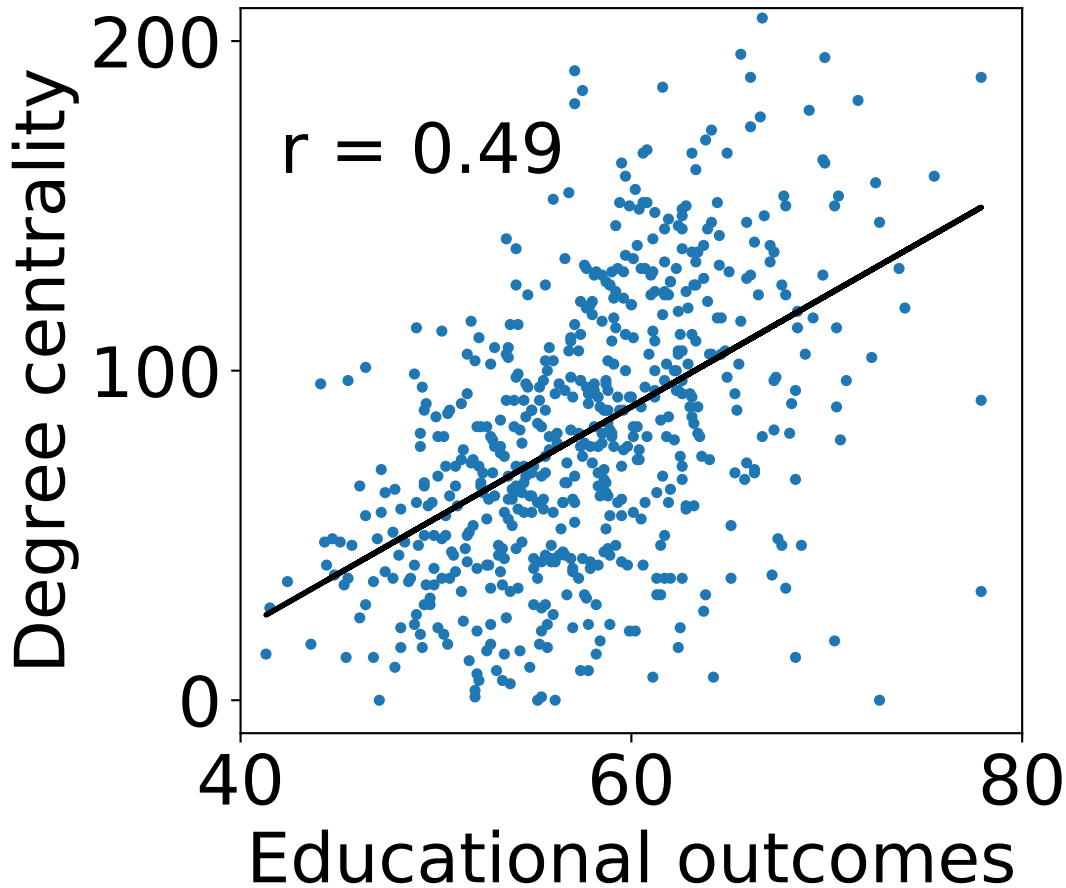

Supplement: S3 Fig — (PDF) [file pone.0217142.s004.pdf]
